# Ponatinib sensitizes myeloma cells to MEK inhibition in the high-risk VQ model

**DOI:** 10.1038/s41598-022-14114-z

**Published:** 2022-06-23

**Authors:** Evan Flietner, Zhi Wen, Adhithi Rajagopalan, Oisun Jung, Lyndsay Watkins, Joshua Wiesner, Xiaona You, Yun Zhou, Yuqian Sun, Brock Kingstad-Bakke, Natalie S. Callander, Alan Rapraeger, M. Suresh, Fotis Asimakopoulos, Jing Zhang

**Affiliations:** 1 McArdle Laboratory for Cancer Research, University of Wisconsin-Madison, Room 7453, WIMR II, 1111 Highland Avenue, Madison, WI 53705 USA; 2 Department of Pathology and Laboratory Medicine, University of Wisconsin School of Medicine and Public Health, University of Wisconsin-Madison, Madison, WI 53705 USA; 3 Center for Precision Medicine Research and Integrated Research and Development Laboratories, Marshfield Clinic Research Institute, Marshfield, WI 54449 USA; 4 Department of Human Oncology, School of Medicine and Public Health, University of Wisconsin-Madison, 1111 Highland Avenue, Madison, WI 53705 USA; 5 Department of Biochemistry, University of Wisconsin-Madison, Madison, WI 53706 USA; 6 Department of Biology, College of Agricultural and Life Sciences, University of Wisconsin-Madison, Madison, WI 53706 USA; 7 Department of Pathobiological Sciences, University of Wisconsin-Madison, Madison, WI USA; 8 Division of Hematology/Oncology, Department of Medicine, UW Comprehensive Cancer Center, University of Wisconsin-Madison, Madison, WI 53705 USA; 9 Division of Blood and Marrow Transplantation, Department of Medicine, University of California-San Diego, La Jolla, CA 92093 USA

**Keywords:** Myeloma, Cancer models, Cancer therapeutic resistance, Chemotherapy, Targeted therapies

## Abstract

Multiple myeloma (MM) is a malignant plasma cell cancer. Mutations in RAS pathway genes are prevalent in advanced and proteasome inhibitor (PI) refractory MM. As such, we recently developed a VQ MM mouse model recapitulating human advanced/high-risk MM. Using VQ MM cell lines we conducted a repurposing screen of 147 FDA-approved anti-cancer drugs with or without trametinib (Tra), a MEK inhibitor. Consistent with its high-risk molecular feature, VQ MM displayed reduced responses to PIs and de novo resistance to the BCL2 inhibitor, venetoclax. Ponatinib (Pon) is the only tyrosine kinase inhibitor that showed moderate MM killing activity as a single agent and strong synergism with Tra in vitro. Combined Tra and Pon treatment significantly prolonged the survival of VQ MM mice regardless of treatment schemes. However, this survival benefit was moderate compared to that of Tra alone. Further testing of Tra and Pon on cytotoxic CD8^+^ T cells showed that Pon, but not Tra, blocked T cell function in vitro, suggesting that the negative impact of Pon on T cells may partially counteract its MM-killing synergism with Tra in vivo. Our study provides strong rational to comprehensively evaluate agents on both MM cells and anti-MM immune cells during therapy development.

## Introduction

Multiple myeloma (MM) is a plasma cell malignancy, representing 10% of hematological cancers and about 2% of all new cancer diagnoses (SEER 2021). Although many patients have benefitted from the introduction of immunomodulatory drugs (IMiDs), proteasome inhibitors (PIs), and monoclonal antibodies, most patients are eventually refractory to these treatments^1^. Mutations in RAS pathway genes (e.g. *NRAS*, *KRAS*, and *BRAF*) are particularly prevalent among IMiD and/or PI refractory patients: 72% of them harbor mutations in one or more of these genes^2^. As such, we recently developed and characterized a MM mouse model, which is driven by two frequent genetic events identified in human MM, namely *MYC* overexpression and oncogenic *Nras*^Q61R^ (called VQ model)^3^. VQ MM mice fully recapitulate the biological and clinical features of human high-risk MM, including hyperproliferation, hyperactivation of MEK/ERK and AKT pathways downstream of RAS, extramedullary MM dissemination, upregulation of PD-1 and TIGIT immune checkpoint pathways, exhaustion of CD4^+^ and CD8^+^ T cells, and expression of the human UAMS-70 high-risk gene signature^3^. These MM phenotypes are serially transplantable in syngeneic recipients. Among the multiple VQ lines we characterized, VQ-D1 recipients have the longest survival, and their myeloma cells predominantly grow in the bone marrow and spleen. By contrast, VQ-D2 recipients have the shortest survival, and their myeloma cells are primarily localized in the spleen and lymph nodes. We also derived two cell lines, VQ 4935 and VQ 4938, from primary VQ-D2 myeloma cells for preclinical studies in vitro^3^. In this study, we aim to develop novel targeted therapies using VQ MM cell lines and validate them in recipient mice transplanted with primary VQ-D1 MM cells.

## Results

### Re-purposing screen identifies de novo resistance of VQ MM cells to the BCL-2 inhibitor venetoclax

We previously showed that an FDA-approved MEK inhibitor, trametinib (Tra), killed MM cells in a dose-dependent manner and downregulated surface PD-L1 expression in vitro^3^. In VQ-D1 MM recipient mice, Tra reversed exhausted cytotoxic CD8^+^ T cell phenotypes (Figure S1) and prolonged their survival^3^. Therefore, we sought to identify new MEK inhibition-based combination therapies utilizing two VQ myeloma cell lines. A high-throughput screening assay was developed in which VQ 4938 cells were cultured in 384-well plates for 48 h and cell viability was measured using the CellTiter-Glo luminescence assay. Cells treated with Tra served as a positive control for the assay, with DMSO treated cells as the negative control. Z’ factor for the assay was consistently greater than 0.50, indicating assay reproducibility and consistency^4^.

**Figure 1:**
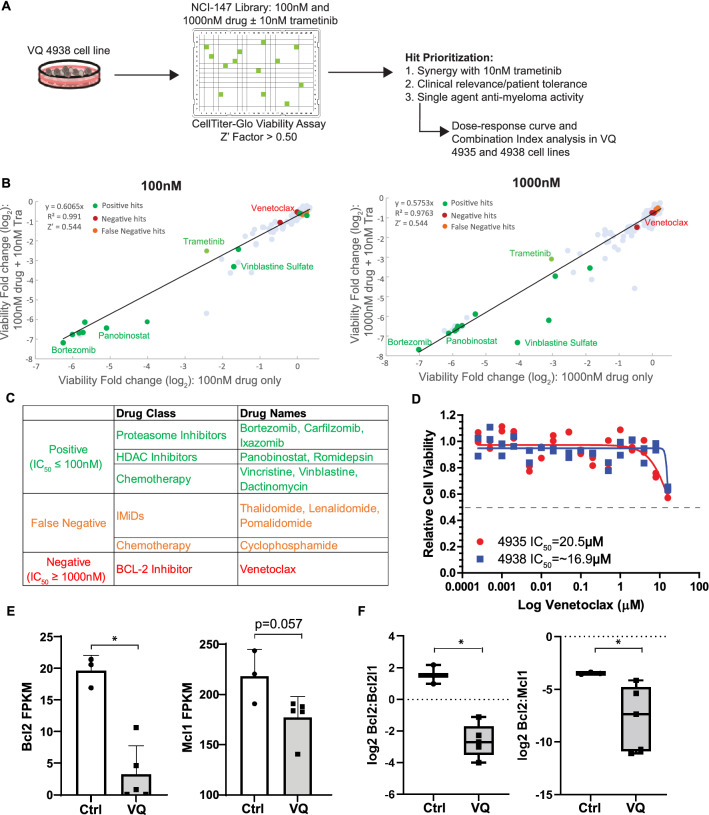
Re-purposing screen identifies de novo resistance of VQ MM cells to venetoclax. (**A**) Scheme of drug screening procedure against VQ myeloma cells. (**B**) AOD IX screening results for compounds at 100 nM and 1000 nM concentration alone or in the presence of 10 nM Trametinib. Results are plotted as Log2 fold change in viability relative to DMSO-treated control wells as measured by CellTiter-Glo Luminescent Assay after 48 h of treatment. Notable compounds are highlighted-see accompanying table in (**C**). (**C**) Table detailing selected positive, false negative, and true negative hits from the AOD IX library as highlighted in (**B**). (**D**) VQ 4935 and 4938 cells were treated with the indicated concentration of venetoclax for 48 h. Relative viability to DMSO treated control was then measured using the CellTiter-Glo assay. IC_50_ values were calculated by logistic regression using the GraphPad Prism software. (**E**) Transcript levels of anti-apoptotic genes Bcl2 and Mcl1 in CD138^+^ B220^-^ cells from control bone marrow (BM) or VQ recipient BM. FPKM, Fragments Per Kilobase of transcript per Million mapped reads. (**F**) Ratios of Bcl2:Bcl2l1 and Bcl2:Mcl1 gene expression levels. Results are presented as mean + SD. *p < 0.05.

To expediate clinical testing, we initially focused on combining Tra with a library of 147 FDA-approved anti-cancer drugs provided by the National Cancer Institute (AOD IX panel). VQ 4938 cells were treated with the AOD IX panel drugs at concentrations of 100 nM and 1000 nM in the presence or absence of 10 nM Tra (Fig. 1A). Viability was measured as the relative change in luminescence compared to DMSO treated wells. Of note, 10 nM Tra alone led to ~ 50% viability relative to DMSO control. Viability fold change of anti-cancer drug alone (X axis) versus viability fold change when the drug was combined with 10 nM Tra (Y axis) was then plotted and analyzed via linear regression (Fig. 1B). Area under the curve represents increased efficacy of compounds when combined with Tra.

The AOD IX panel includes many drugs approved for MM treatment. Based on their initial screening results as single agents (Table S1) and the knowledge of drug actions, these compounds were classified as positive, false negative, or negative (Fig. 1C). False negative group included cyclophosphamide, a pro-drug that needs to be metabolized by the liver to be active in vivo^5^, and IMiDs (e.g. lenalidomide), which are known to be ineffective against murine cells due to the species difference at the cereblon (Crbn) codon 391^6^. Our in vitro validation of VQ response to lenalidomide (Figure S2) is consistent with its in vivo testing in Vk*MYC mice^7^.

Interestingly, VQ 4938 cells showed de novo resistance to venetoclax, with an IC_50_ > 1000 nM in the primary screen (Table S1). We subsequently validated this result using two VQ cell lines and a broad range of drug concentrations (Fig. 1D). Consistent with our primary screen result, the IC_50_ was not reached at 16 µM in both VQ cell lines and estimated to be ~ 20 µM. Venetoclax is considered one of the few targeted therapies for MM patients with t(11;14) translocations and/or high *BCL2:BCL2L1* and *BCL2:MCL1* gene expression ratios^8^. Therefore, we investigated the expression levels of *Bcl2* and *Mcl1* as well as *Bcl2* ratios to *Bcl2l1* and *Mcl1* in VQ MM cells. Not surprisingly, RNA-Seq analysis of primary VQ MM cells and control plasma cells^3^ showed that *Bcl2* and *Mcl1* expression levels (Fig. 1E) and both expression ratios (Fig. 1F) were lower in VQ MM cells than those in control plasma cells. Together, our data suggest that VQ MM cells may not depend on *Bcl2* for survival and are thus de novo resistant to venetoclax.

### Proteasome inhibitors show limited efficacy in the VQ model

The Positive group included PIs (e.g. bortezomib [Btz] and carfilzomib [Cfz]), HDAC inhibitors (e.g. panobinostat and romidepsin), and several chemotherapy agents (e.g. vinblastine sulfate and vincristine sulfate) (Fig. 1C and Table S1). Again, these results were validated using both VQ 4935 and 4938 cell lines in dose response tests (Figures S3, S4A, and S4D). In comparison to human myeloma cell lines^9^, both VQ MM cell lines displayed increased resistance to Btz and Cfz based on their IC_50_ values (~ 9 nM and ~ 60–70 nM respectively, Figures S4A and S4D). This is in line with clinical data showing patients with *NRAS* mutations have reduced Btz sensitivity^10^. Because PIs are used in all lines of MM treatment, we further explored them in vivo. In our previous study^3^, we used Btz in the VQ model as a single agent following a treatment scheme established with the Vk*Myc model^7^. However, a significant proportion of treated mice died soon after the treatment, suggesting that VQ MM mice may not tolerate this treatment scheme very well. Therefore, we adjusted it based on the current clinical practice in human patients and found that this revised scheme showed transient effectiveness in controlling VQ growth in vivo (Figure S4B) and provided a moderate but significant increase in survival (Figure S4C).

To further boost the survival benefit, we used Cfz as part of a combination therapy regimen with dexamethasone (Dex), Tra, and GSK525762 (GSK), a pan-BET inhibitor^11^. We previously showed that combined Tra and GSK prolonged the survival of VQ MM mice better than single agents alone^3^. In this new combination treatment, Cfz and Dex were administered once a week for two weeks, followed by one week of daily treatment with Tra and GSK. Although combo therapy slowed VQ growth after the first treatment cycle (Figure S4E), it did not significantly prolong the survival of VQ-bearing mice (Figure S4F). Overall, our data show that PIs only provide short-term disease control in the VQ model.

### Combination trametinib and ponatinib treatment are synergistic against VQ myeloma cells in vitro

**Figure 2:**
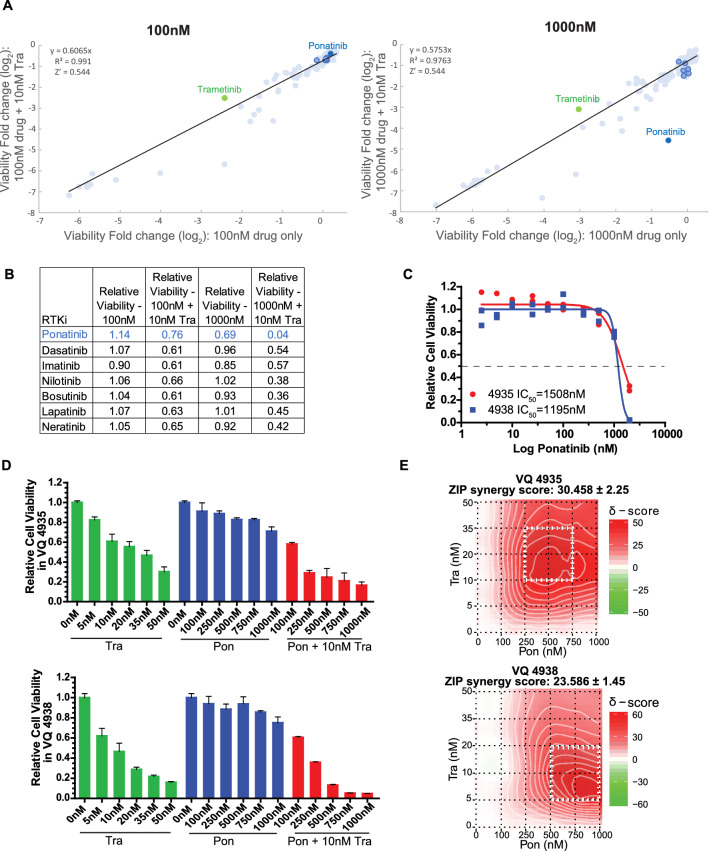
Ponatinib, but not other TKIs, synergizes with trametinib in vitro. (**A**) Relative viability results for tyrosine kinase inhibitors (TKIs) from AOD IX panel at 100 nM and 1000 nM concentration alone or in the presence of 10 nM trametinib, as in Fig. 1A. (**B**) Screening results of TKIs as single agents and with 10 nM trametinib. Trametinib and Ponatinib are highlighted as in (**A**). Of note, 10 nM trametinib yielded ~ 50% viability relative to DMSO control. (**C–E**) VQ 4935 and 4938 cells were treated with the indicated concentrations of two compounds for 48 h. Relative viability to DMSO treated control was then measured using the CellTiter-Glo assay. (**C**) Dose–response results for ponatinib against VQ 4935 and 4938 cell lines. IC_50_ values were calculated by logistic regression using the GraphPad Prism software. (**D**) Selected viability results for combination treatment of trametinib (Tra) and ponatinib (Pon) against VQ 4935 and 4938 cells. (**E**) ZIP synergy plots of Tra and Pon in VQ 4935 and 4938 cells. Zip Synergy scores were generated using the SynergyFinder online tool.

Screening of the AOD IX panel identified 1000 nM ponatinib (Pon) as having high synergy with Tra against VQ myeloma cells (Fig. 2A,B). Pon is a multi-tyrosine kinase inhibitor (TKI) currently approved for second-line treatment of chronic myeloid leukemia and Philadelphia chromosome-positive acute lymphoblastic leukemia^12^. Interestingly, no significant efficacy was observed for other TKIs as single agents or in combination with Tra (Fig. 2A,B). Although dose–response testing of VQ 4935 and 4938 cell lines confirmed that Pon had limited effect as a single agent (Fig. 2C), it showed strong synergy with Tra against both VQ cell lines based on ZIP delta score analysis^13^ (Fig. 2D,E) and Combination Index calculation^14^ (Figure S5). Of note, this synergy appeared to be more prominent at higher concentrations of Pon (> 250 nM).

We noticed that unlike other TKIs in the AOD IX panel, Pon is a potent pan-fibroblast growth factor receptor (FGFR) inhibitor with IC_50_ values < 100 nM^15^. In human MM, FGFR3 is overexpressed in the t(4;14) high-risk subset as the translocation places FGFR3 expression under the control of the heavy chain immunoglobulin promoter on chromosome 14^16^. However, parental VQ-D2 cells show little to no expression of FGFR1-4 at the mRNA level, comparable to control plasma cells (Figure S6A) and do not harbor any mutations in FGFR1-4 either (our unpublished results). To determine if FGFR inhibition plays a role in Pon efficacy, we next tested the FGFR1 inhibitor sorafenib^17^, the FGFR1-3 inhibitor pazopanib^18^, and the pan-FGFR inhibitors dovitinib and lenvatinib^19,20^ in our VQ MM cell lines. Similar as other TKIs tested in the AOD IX panel, none of the FGFR inhibitors displayed single agent efficacy at 100 nM and 1000 nM or in combination with Tra (Figure S6B), suggesting that the efficacy of Pon in VQ MM cells is not primarily mediated through FGFR signaling.

To further explore the efficacy of Pon and the synergy between Tra and Pon in vitro, we extended the drug treatment to several human myeloma cell lines (HMCLs), including OPM2 with t(4;14) translocation, MM.1S with oncogenic *KRAS* mutation, H929 with t(4;14) translocation and oncogenic *NRAS* mutation, and Delta47 without either event^21–23^. Interestingly, regardless of t(4;14) status, OPM2 and MM.1S were more responsive to Pon with an IC_50_ at ~ 1.4–2 µM (Fig. S7A). These results are consistent with those from VQ MM cell lines.

When HMCLs were treated with combined Tra and Pon using the same range of concentrations as in VQ MM cell lines, we only observed a moderate additive effect in OPM2 and MM.1S cell lines (Fig. S7B), which may result from the minimal response of these HMCLs to 10–50 nM Tra.

### Combination trametinib and ponatinib significantly prolongs survival of VQ mice in two different treatment regimens

**Figure 3:**
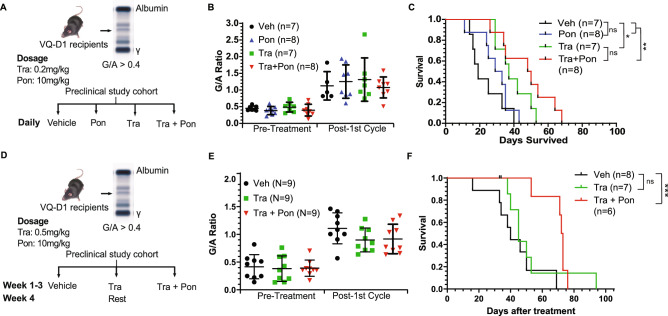
Combination trametinib and ponatinib treatment prolongs VQ myeloma survival. (**A**) Scheme of pre-clinical treatment groups and in vivo drug dosages. Mice were treated with the indicated compounds daily as described in Materials and Methods. (**B**) Serum protein electrophoresis was performed to quantify the γ-globulin/Albumin (G/A) ratios in VQ recipient mice before treatment and at day 21 of treatment. Note: Two Vehicle-treated recipients were found dead and unable to be analyzed. (**C**) Kaplan–Meier survival curves were plotted against days after treatment. Log-rank test was performed. (**D**) Scheme of pre-clinical treatment groups and in vivo drug dosages. Mice were treated with the indicated compounds in 28-day cycles (3-weeks on and one-week off) as described in Materials and Methods. (**E**) Serum protein electrophoresis was performed to quantify the G/A ratios in VQ recipient mice before treatment and at day 21 of treatment. Note: One Vehicle-treated recipient was found dead and unable to be analyzed. (**F**) Kaplan–Meier survival curves were plotted against days after treatment. Log-rank test was performed. Note: One vehicle-treated animal was euthanized for reasons unrelated to treatment study and was excluded from analysis. *p < 0.05; **p < 0.01; ***p < 0.001.

Because Pon is clinically available as an oral agent and has not been evaluated in MM before, it was of interest to determine its in vivo efficacy alone and in combination with Tra. VQ-D1 MM cells were transplanted into sub-lethally irradiated recipient mice as previously described^3^. Once MM was established, recipients were divided into 4 groups with comparable gamma-globulin to albumin (G/A) ratios and similar complete blood count (CBC) parameters and treated with vehicle, Tra, Pon, and combined Tra and Pon (Figs. 3A and S8). Twenty-one days after treatment, all four groups of mice showed increased but indistinguishable G/A ratios (Fig. 3B) and the overall CBC results were unchanged (Figure S8). Consistent with our in vitro analysis, Pon treatment did not prolong the survival of VQ-D1 MM mice, while both Tra and combined Tra and Pon treatments did (Fig. 3C). Although combination treated mice had the longest overall survival, we did not observe significant difference between Tra- and Tra/Pon-treatment groups.

We subsequently sought to determine if increasing the Tra dosage would significantly prolong the survival of Tra/Pon treated mice. To combat against the potential cumulative toxicity associated with higher Tra dose, we also took the 3-week on and 1-week off schedule as followed in a typical myeloma treatment regimen^1^. In a second in vivo experiment, recipients were divided into 3 groups with comparable G/A and CBC parameters and then treated with vehicle, Tra alone, or combination Tra/Pon in 28-day cycles (Figs. 3D and S9). Once again, no significant difference was observed in G/A ratios between groups after one treatment cycle (Figs. 3E and S9). Interestingly, although no survival benefit was observed with single agent Tra treatment, combo treated mice had significantly prolonged survival compared to the vehicle-treated group (Fig. 3F).

### Ponatinib, but not trametinib, inhibits CD8^+^ T cell proliferation and activation in vitro

**Figure 4:**
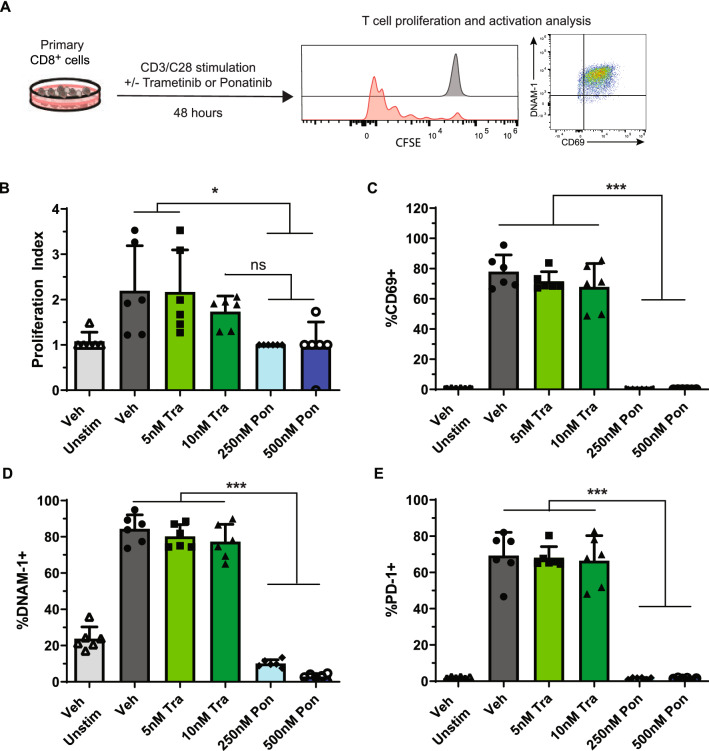
Ponatinib, but not trametinib, blocks CD8^+^ T cell proliferation and activation in vitro. (**A**) Experimental scheme for CD8^+^ T cell proliferation and activation assay. CD8^+^ T cells isolated from the spleens of C57BL/6J mice were stained with CFSE and cultured in the presence of plate-bound α-CD3 and soluble α-CD28, along with the indicated concentrations of trametinib and ponatinib, for 48 h. Cells were then analyzed using flow cytometry. Statistical differences between multiple groups were determined via one-way ANOVA with Tukey’s post-test analysis. (**B**) Proliferation Index was calculated for each group via FCS Express v7.08 software. (**C–E**) Quantification of CD69^+^ (**C**), DNAM-1^+^ (**D**), and PD-1^+^ (**E**) CD8^+^ T cells. Results are presented as mean + SD. *p < 0.05; **p < 0.01; ***p < 0.001.

We investigated if the discrepancy between in vitro and in vivo combo treatment outcomes results from the drug effects on cytotoxic CD8^+^ T cells, which play an important role in anti-MM immunity^24^. To test this idea, CD8^+^ T cells were isolated from spleens of wildtype B6 mice, stained with CFSE dye, and activated via α-CD3/α-CD28 antibodies in the presence of Tra or Pon for 48 h (Fig. 4A). Tra treatment did not cause significant reduction in T cell proliferation as measured by CFSE tracing (Fig. 4B) and T cell activation as demonstrated by surface expression of CD69, DNAM-1, and PD-1 (Fig. 4C–E). By contrast, Pon treatment completely inhibited T cell proliferation and activation (Fig. 4B, C). Our results are consistent with prior studies showing that ponatinib and related BCR-ABL TKIs (e.g. dasatinib, imatinib) impair T cell function and viability in a dose-dependent manner^25–27^.

## Discussion

While the introduction of IMiDs, PIs and monoclonal antibody treatments has revolutionized MM therapy, most patients still develop drug-refractory disease and eventually die of myeloma^1^. RAS pathway hyperactivation is a common molecular event in progressive myeloma, with almost 75% of drug-refractory myeloma patients harboring mutations in *NRAS*, *KRAS*, or *BRAF*^2^. In this study, our group used the recently developed VQ model of high-risk myeloma as a platform to develop new treatment regiments. To assess the effectiveness of existing MM therapies against VQ myeloma and expedite clinical testing, we carried out a re-purposing screen of 147 FDA-approved anti-cancer compounds against VQ cells in vitro (Fig. 1A). We found that VQ cells showed de novo resistance to venetoclax (Fig. 1D), likely owing to low Bcl2:Bcl2l1 and Bcl2:Mcl1 gene expression ratios (Fig. 1F)^8^. In addition, VQ cells showed increased resistance to Btz (Figure S4A) and Cfz (Figure S4D) compared to human MM cell lines in vitro^9^. Limited response to PI treatment was also observed in vivo, either as single agent (Figure S4C) or as part of multi-drug treatment regimen (Figure S4F). This is not altogether unexpected, as resistance to single agent Btz has been observed in patients harboring *NRAS* mutations^10^.

As a single agent, Tra displays dual effects on MM cells and T cells. Tra kills MM cells in a dose-dependent manner and downregulates surface PD-L1 expression in vitro^3^. Tra treatment of purified splenic CD8^+^ T cells in vitro did not significantly impact their proliferation (Fig. 4B) and activation (Fig. 4C–E). These in vitro T cell results are consistent with our prior study that downregulation of RAS/ERK signaling in *Kras*^*-/-*^ T cells does not affect CD8^+^ T cell-mediated anti-leukemia activity in vivo^28^. In VQ-D1 MM recipients, Tra reversed exhausted cytotoxic CD8^+^ T cell phenotypes (Figure S1) and prolonged their survival^3^. MEK inhibition has previously been shown to have pro-CD8^+^ T cell effects in oncogenic Kras-driven colorectal cancer^29^. In the context of the Kras^G12D^ CT26 model, multiple groups found that MEK inhibition reduces PD-1 expression and prevents apoptosis of antigen-experienced CD8^+^ T cells^29,30^. Of note, low dose, continuous Tra treatment (Fig. 3C) worked better than high dose, on/off Tra treatment in vivo (Fig. 3F). This observation is consistent with cancer patients treated with Tra^31^.

Interestingly, among all the TKIs in the AOD IX panel and other FGFR inhibitors, only Pon showed moderate cell killing at 1 µM as single agent (Fig. 2B) and strong synergism with Tra at concentrations above 250 nM (Fig. 2D, E). Our data suggest that the efficacy of Pon in VQ MM cells does not primarily come from its inhibition of FGFRs. Rather, Pon may be targeting other tyrosine kinases due to its multi-RTK inhibitory function. This possibility is supported by our results from HMCLs, which responded to Pon regardless of their t(4,14) status (Fig. S7). Alternatively, Pon is a relatively more potent TKI compared to others; the clinical dose of Pon is indeed lower than those of other TKIs^32^. However, considering 1 µM is a fairly high concentration in vivo, we did not pursue other TKIs at higher concentrations.

Synergism between MEK and FGFR inhibition has previously been established in the context of KRAS mutant lung cancer: a shRNA screen of Tra-treated H23 KRAS^G12C^ lung cancer cells identified FGFR1 signaling as compensatory for MEK inhibition^33^. H23 cells were highly susceptible to Tra and Pon combination treatment^33^, similar as what we observed in the VQ model. However, in a phase I clinical of KRAS mutant non-small cell lung cancer patients, it was found that combination Tra/Pon treatment was associated with cardiovascular (CV) and bleeding toxicities^34^. CV side effects of this combo treatment are not unsurprising, as Pon was temporarily withdrawn by the US Food and Drug Administration in 2013 for heart failure and other CV side effects before being returned with additional safety warnings and restrictions^35^.

Despite the strong synergistic effects of Tra + Pon in vitro, the in vivo effect was only moderately better than Tra alone (Fig. 3). We postulated that the anti-myeloma effects of combination Tra/Pon treatment were abrogated by their unfavorable impacts on non-myeloma cells, such as myeloma killing cytotoxic CD8^+^ T cells. Indeed, Pon, but not Tra, prevented CD8^+^ T cell proliferation and activation (Fig. 4B–E). Our data suggest that the negative impact of Pon treatment on T cells may counteract its MM-killing synergism with Tra in vivo. It is possible that other yet unidentified negative effects of these drugs exist. The complexity of MM in vivo warrants a comprehensive evaluation of agents on MM cells and other important anti-MM immune cells during therapy development. Nonetheless, our data support investigation of combination Tra/Pon treatment in relapsed/refractory MM patients with pre-existing lymphopenia.

## Methods

### Mice

CD45.1^+^ transplant recipients were purchased from Jackson Laboratory (stock # 002014) and maintained at Biotron Animal Research Services facility, University of Wisconsin-Madison. Mice were 8–14 weeks old at time of transplant and male and female mice were used in approximately equal proportion. All animal experiments were conducted in accordance with the Guide for the Care and Use of Laboratory Animals and approved by an Animal Care and Use Committee at UW-Madison. The program is accredited by the Association for Assessment and Accreditation of Laboratory Animal Care. All animal experiments in this study are reported in accordance with ARRIVE guidelines (https://arriveguidelines.org).

### Drug screening of VQ cell lines

The Approved Oncology Drugs (AOD) IX drug panel was provided to the University of Wisconsin-Madison (UW-Madison) by the National Cancer Institute’s Division of Treatment & Diagnosis. Dilution and preparation of the AOX IX panel in 384 well plates was carried out by UW-Madison’s Small Molecule Screening Facility (SMSF). The FGFR inhibitors sorafafenib, dovitinib, pazopanib HCL, and Lenvatinib were kindly provided by the SMSF’s inhibitor library.

VQ 4935 and 4938 cell lines were cultured in IMDM (Corning, 15-016-CM) containing 10% FBS (Gibco, Cat No. 16000), 1X antibiotics, and 10 ng/ml human recombinant IL6 (PeproTech, 200-06) at 37 °C. They were seeded 50 uL/well at a density of 5 × 10^5^ cells/ml in a 384-well plate using a MicroFlo Select Reagent Dispenser (BioTek). 48 h later, cell viability was evaluated using CellTiter-Glo assay (Cat No. G7573, Promega). Chemiluminescence was measured using an ENSPIRE Plate Reader (Perkin Elmer). Z’ Factor was calculated as previously described^4^.

IC_50_ values were calculated via logistic regression with variable slope using GraphPad Prism v9.2.0 software.

### Drug combination studies

Drug combination studies were set up using a 5 × 5 matrix design around IC_50_ of each drug in VQ 4935 and 4938 cell lines. Synergy was calculated using ZIP delta score via the SynergyFinder online tool (https://synergyfinder.fimm.fi)^13^. Combination Index scores were calculated using Compusyn v1.0 software as previously described^14^.

### Human myeloma cell line (HMCL) culture and drug screening

OPM2, Delta-47, MM.1S and H929 were cultured per ATCC recommendations at 37 °C, 5% CO_2_. Cells were cultured in RPMI-1640 media (Hyclone, cat# SH30027FS) supplemented with 10% FBS (Sigma, cat# F2442-500ML). H929 cells were also cultured in 1X 2-Mercaptoethanol (GIBCO, cat# 21–985-023). Cells were freshly passaged 24 h prior to drug testing. 100 µl of 0.3 × 10^6^/ml cells were plated per well in 96-well plates with pre-added trametinib or ponatinib. 48 h later, cell viability was evaluated using CellTiter-Glo assay (Cat No. G7573, Promega). Chemiluminescence was measured using an OMEGA microplate reader (BMG Labtech).

### Transplantation of myeloma cells

Donor cells from two moribund VQ-D1 MM bearing mice were pooled equally and resuspended in 100 μl of PBS containing 2% mouse serum (Jackson ImmunoResearch, 015-000-120). Eight- to fourteen-week-old CD45.1^+^ recipient mice were sub-lethally irradiated at 4.0 Gy using an X-RAD 320 Irradiator (Precision X-Ray Inc.) and transplanted with 5 × 10^5^ of donor cells via intracardiac injection.

### Serum protein electrophoresis (SPEP)

Mice were retro-orbitally bled with plain micro hematocrit tubes (Bris, ISO12772). Blood samples were spun in microtainer tubes (BD, 365967) at 2000×*g* for 10 min to collect serum. Serum was loaded into Hydragel agarose gel (Sebia, 4140) and processed using the Hydrasys instrument (Sebia) following the manufacturer’s instruction. The processed film was scanned and pixel density of Albumin and γ-globulin bands were quantified using Adobe Photoshop.

### Complete blood count

Peripheral blood samples were collected via retro-orbital bleeding and analyzed with a Hemavet 950FS (Drew Scientific).

### Small compound treatment

For in vivo bortezomib treatment, bortezomib (Selleck) was dissolved in sterile PBS and administered at 0.5 mg/kg twice a week for four weeks via intra-peritoneal (IP) injection.

For in vivo treatment of carfilzomib, dexamethasone, trametinib, and GSK525762, carfilzomib (Selleck) was dissolved in sterile PBS and administered at 16 mg/kg once a week via IP injection for two weeks. Dexamethasone (Selleck) was dissolved in sterile PBS and administered at 1 mg/kg once a week via IP injection for two weeks. Trametinib (Chemitek) was dissolved in 0.5% hydroxypropylmethylcellulose (Sigma) and 0.2% Tween-80 (Sigma) in distilled water (pH 8.0) and given orally at 0.5 mg/kg every morning for one week. GSK525762 (Chemitek) was dissolved in 1% methylcellulose (Sigma) containing 0.2% SDS and given orally at 15 mg/kg every evening for one week.

For in vivo treatment of trametinib and ponatinib, both compounds were dissolved in 0.5% hydroxypropylmethylcellulose (Sigma) and 0.2% Tween-80 (Sigma) in distilled water (pH 8.0) and administered at 0.2 mg/kg and 10.0 mg/kg respectively, via oral gavage daily. In a second treatment experiment, trametinib and ponatinib were dissolved in 0.5% hydroxypropylmethylcellulose (Sigma) and 0.2% Tween-80 (Sigma) in distilled water (pH 8.0) and administered at 0.5 mg/kg and 10.0 mg/kg, respectively, in 28 day cycles with 21 days of treatment followed by 7 days of rest.

Mice were not allocated to treatment groups in a blinded manner but were instead allocated so that G/A and CBC parameters were statistically similar between each group (One-way Analysis of Variance with Tukey–Kramer test). Small compounds were not administered to animals in a blinded manner due to necessary daily preparation of working concentrations for treatment. Animal care staff were blinded to experimental groups during animal assessment. Post-experiment data analysis was not blinded.

### CD8^+^ T cell activation assay

CD8^+^ T cells were purified from total splenocytes of 8–14 weeks old C57BL/6J mice using the Mouse CD8α^+^ T Cell Isolation Kit (Miletnyi Biotec, 130-104-75) and labeled with CFSE (eBioscience, 65-850-84) as described^36^. CD8^+^ T cells were cultured in RPMI-1640 (Corning, 15-041-CV) containing 10% FBS, 1X Antibiotics, 1X GlutaMAX (Gibco, 35050061), 1X MEM non-essential amino acids solution (Gibco, 11140050), 1 mM Sodium Pyruvate (Gibco, 11360070), and 50 µM 2-Mercaptoethanol (Gibco, 21985-23). T cells were activated in the presence or absence of trametinib and ponatinib using plate-bound α-CD3 (eBioscience, 17A2; 50 µL of 10 µg/mL solution incubated at 4 °C overnight) and soluble α-CD28 (eBioscience, 17A2; 5 µg/mL) for 48 h prior to analysis.

### Flow cytometric analysis of hematopoietic tissues

Flow cytometric analysis of surface antigens on hematopoietic cells was performed as previously described^37^. Stained cells were analyzed on a LSRII Fortessa (BD Biosciences). Directly conjugated antibodies specific for the following mouse surface antigens were purchased from Biolegend unless specified: CD3(17A2), CD4 (eBioscience, GK1.5), CD8 (eBioscience, 53-6.7), CD62L(MEL-14), CD44(IM7), PD1 (29F.1A12), TIGIT(GIGD7), LAG3(C9B7W), DNAM-1(TX42.1), CD69 (eBioscience, H1.2F3).

### Statistics

For Kaplan–Meier survival curves, survival differences between groups were assessed with the log-rank test, assuming significance at *p* < 0.05. Unpaired, two-way t Test was used to determine significant differences between two groups unless specified. One-way Analysis of Variance with Tukey–Kramer test was used to determine the significance between multiple data sets simultaneously unless specified, assuming significance at *p* < 0.05. Statistical analysis was carried out using GraphPad Prism v9.2.0.

## Supplementary Information

Supplementary Information 1.

Supplementary Information 2.

Supplementary Information 3.

Supplementary Information 4.

Supplementary Information 5.

Supplementary Information 6.

Supplementary Information 7.

Supplementary Information 8.

Supplementary Information 9.

Supplementary Information 10.

Supplementary Information 11.

Supplementary Information 12.

## Data Availability

The datasets generated and/or analyzed during the current study are available from the corresponding author on reasonable request.
