# Supplementary figures and images for: Ponatinib sensitizes myeloma cells to MEK inhibition in the high-risk VQ model
Source: Sci Rep. 2022 Jun 23;12:10616. doi: 10.1038/s41598-022-14114-z (PMC9226136; doi:10.1038/s41598-022-14114-z)

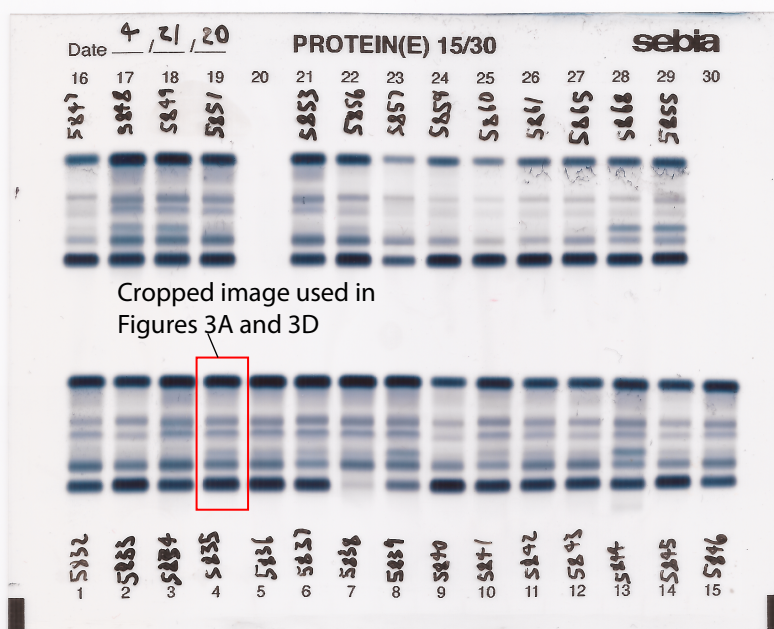

Supplement: Supplementary file 1 — Supplementary Information 1. [file 41598_2022_14114_MOESM1_ESM.pdf]

A

tSNE analysis of  
VQ Bone Marrow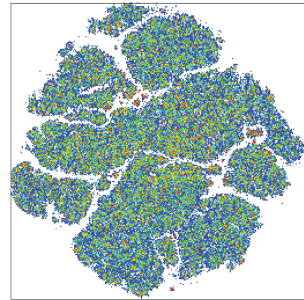Gated  
CD8+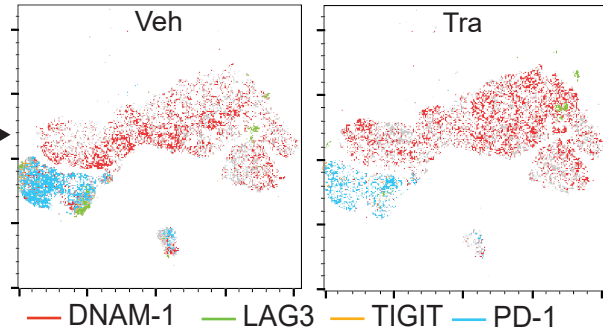

B

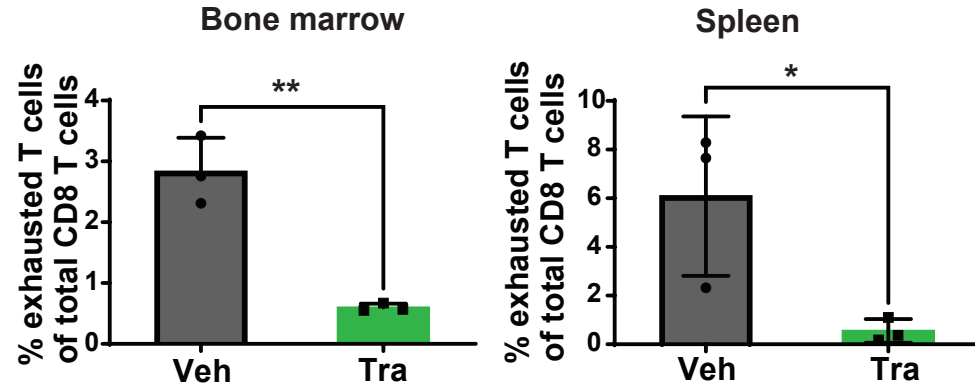

Supplement: Supplementary file 2 — Supplementary Information 2. [file 41598_2022_14114_MOESM2_ESM.pdf]

Figure S2-Zhang

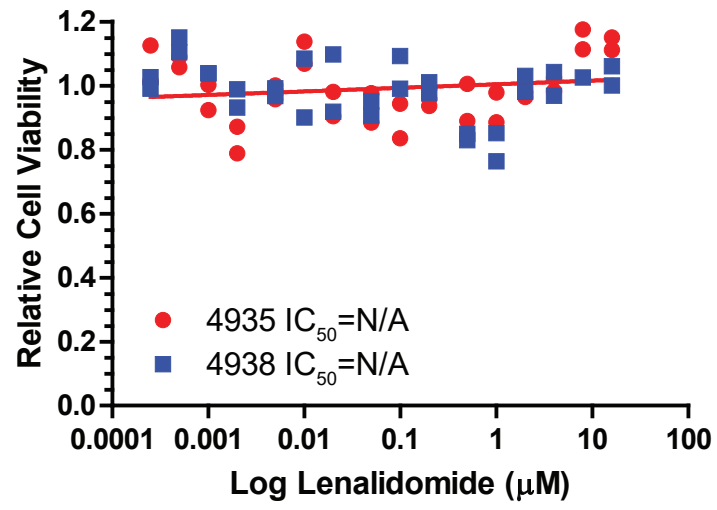

Supplement: Supplementary file 3 — Supplementary Information 3. [file 41598_2022_14114_MOESM3_ESM.pdf]

A

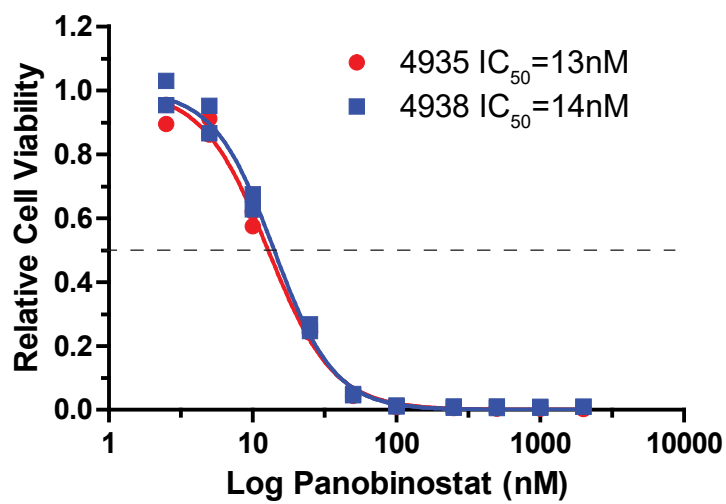

B

Figure S3-Zhang

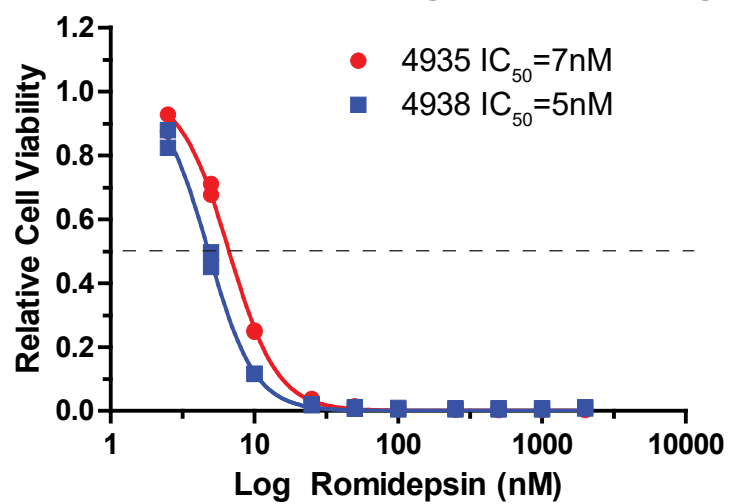

C

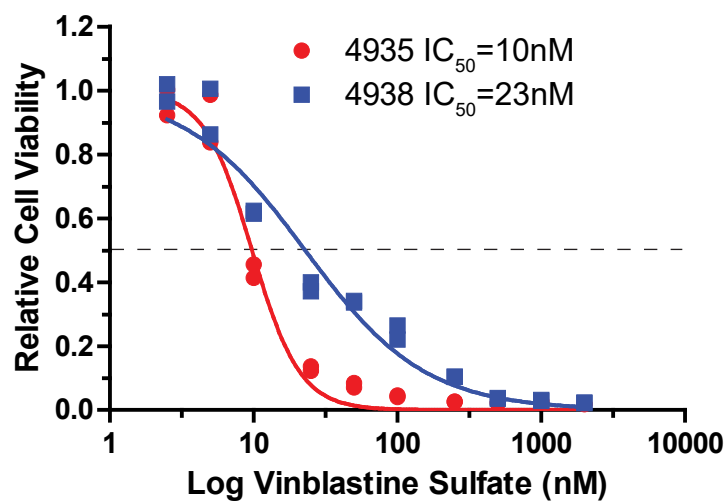

D

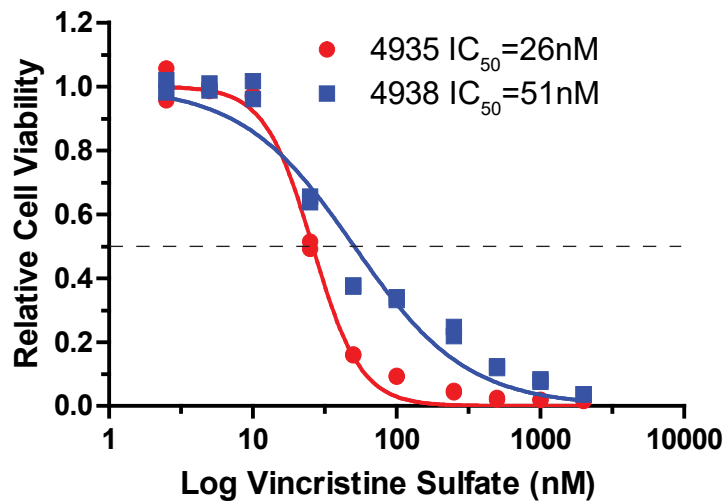

Supplement: Supplementary file 4 — Supplementary Information 4. [file 41598_2022_14114_MOESM4_ESM.pdf]

Figure-S4, Zhang

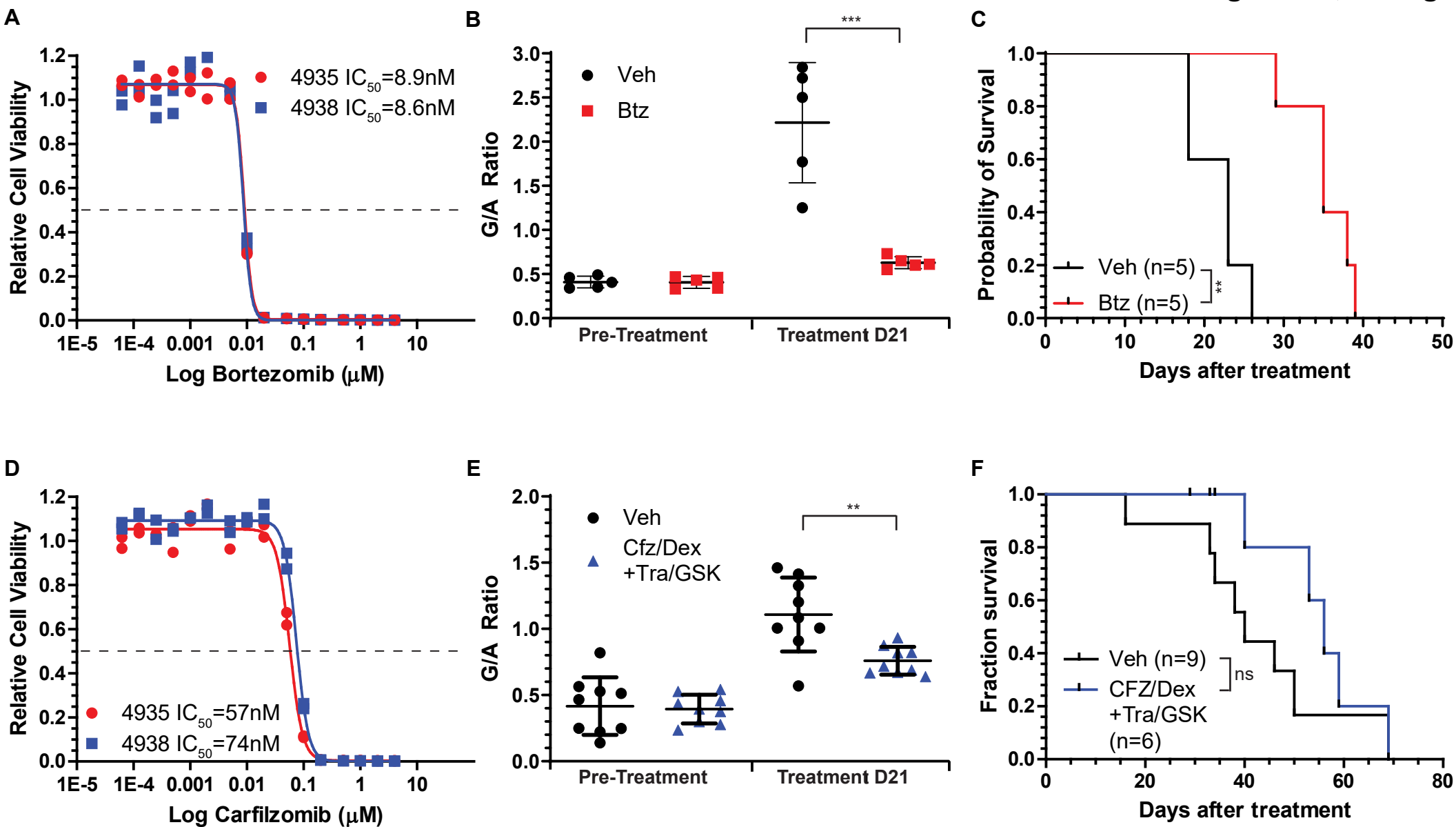

Supplement: Supplementary file 5 — Supplementary Information 5. [file 41598_2022_14114_MOESM5_ESM.pdf]

A

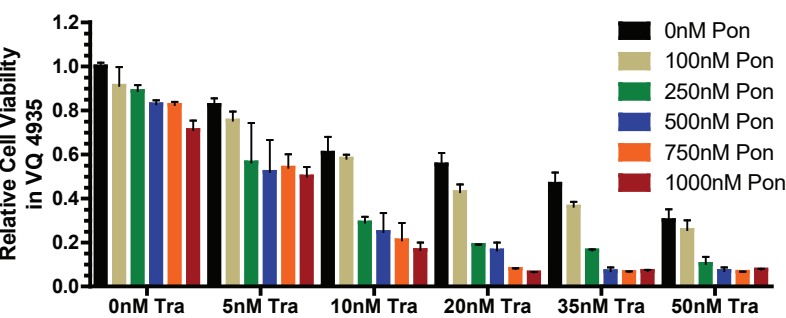

| Trametinib | Ponatinib |       |       |       |       |       |
|------------|-----------|-------|-------|-------|-------|-------|
|            | CI: 4935  | 100   | 250   | 500   | 750   | 1000  |
| 5          |           | 0.833 | 0.332 | 0.306 | 0.378 | 0.334 |
| 10         |           | 0.638 | 0.166 | 0.134 | 0.107 | 0.078 |
| 20         |           | 0.616 | 0.172 | 0.143 | 0.058 | 0.045 |
| 35         |           | 0.810 | 0.249 | 0.086 | 0.081 | 0.088 |
| 50         |           | 0.660 | 0.194 | 0.123 | 0.115 | 0.136 |

B

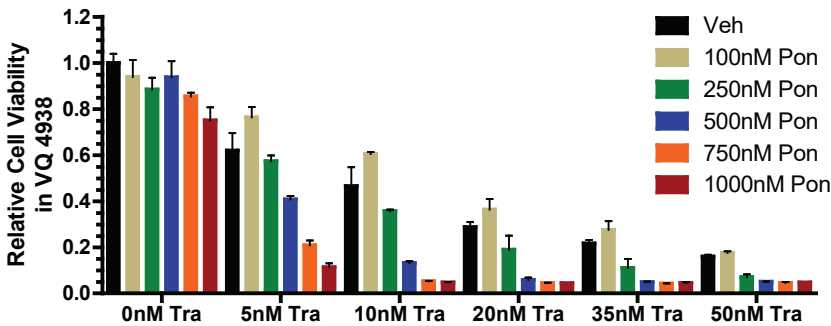

| Trametinib | Ponatinib |       |       |       |       |       |
|------------|-----------|-------|-------|-------|-------|-------|
|            | CI: 4938  | 100   | 250   | 500   | 750   | 1000  |
| 5          |           | 2.142 | 0.847 | 0.413 | 0.146 | 0.067 |
| 10         |           | 1.893 | 0.642 | 0.160 | 0.054 | 0.049 |
| 20         |           | 1.318 | 0.507 | 0.121 | 0.091 | 0.091 |
| 35         |           | 1.491 | 0.451 | 0.171 | 0.147 | 0.163 |
| 50         |           | 1.145 | 0.381 | 0.255 | 0.232 | 0.244 |

Supplement: Supplementary file 6 — Supplementary Information 6. [file 41598_2022_14114_MOESM6_ESM.pdf]

A

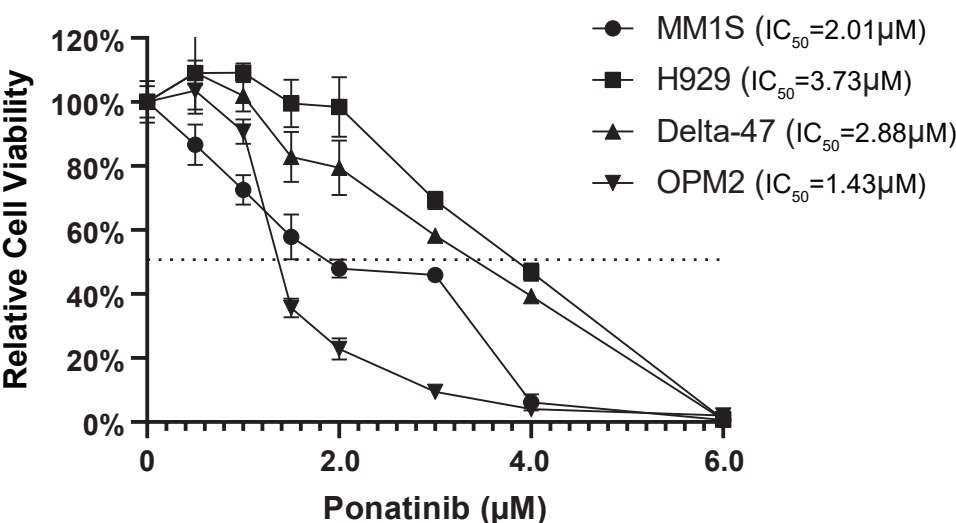

B

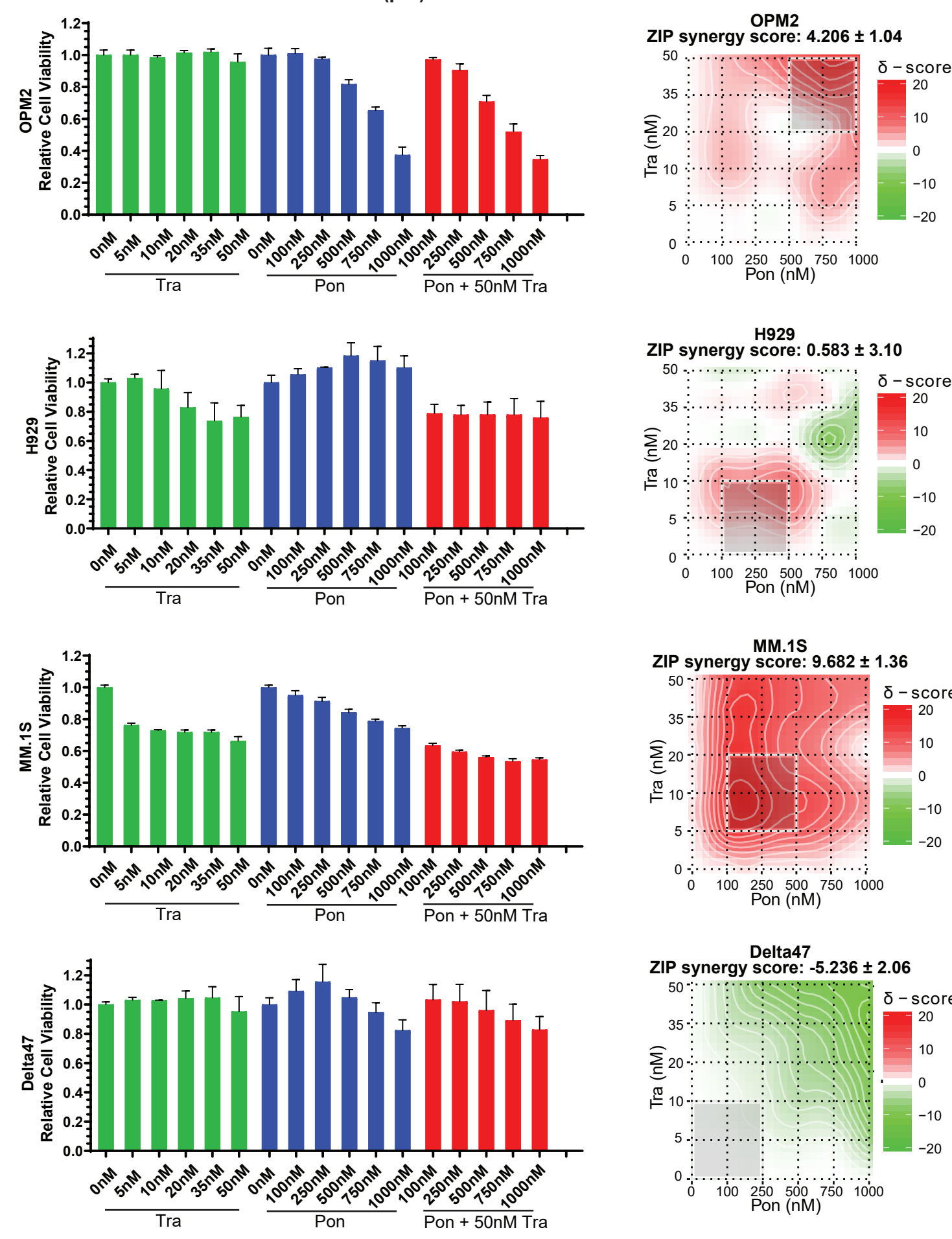

Supplement: Supplementary file 8 — Supplementary Information 8. [file 41598_2022_14114_MOESM8_ESM.pdf]

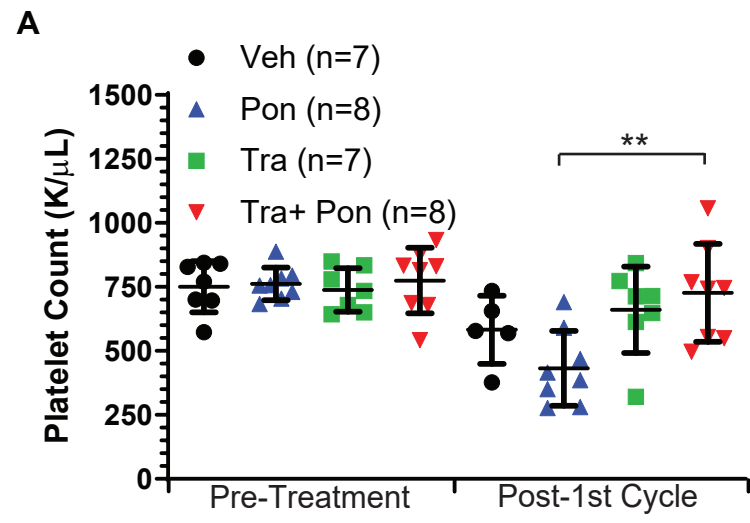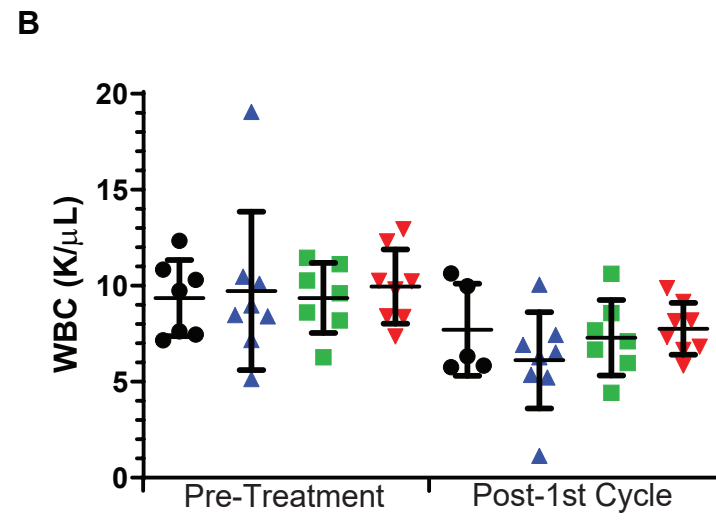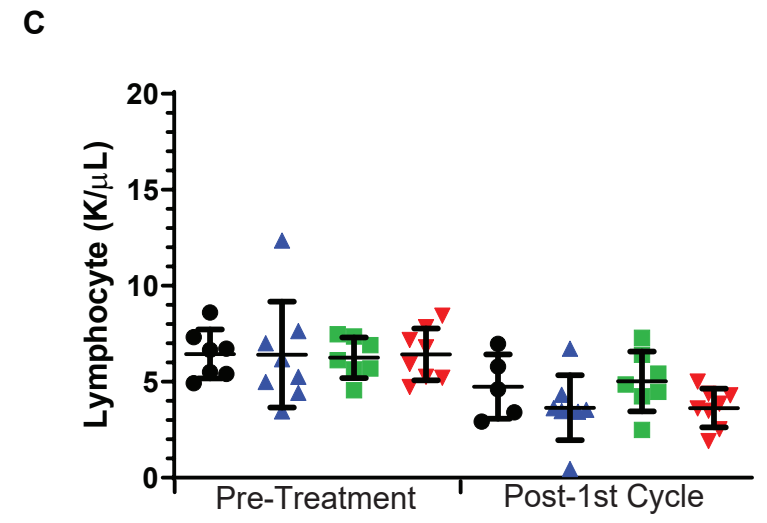

Supplement: Supplementary file 9 — Supplementary Information 9. [file 41598_2022_14114_MOESM9_ESM.pdf]

Figure S9-Zhang

A

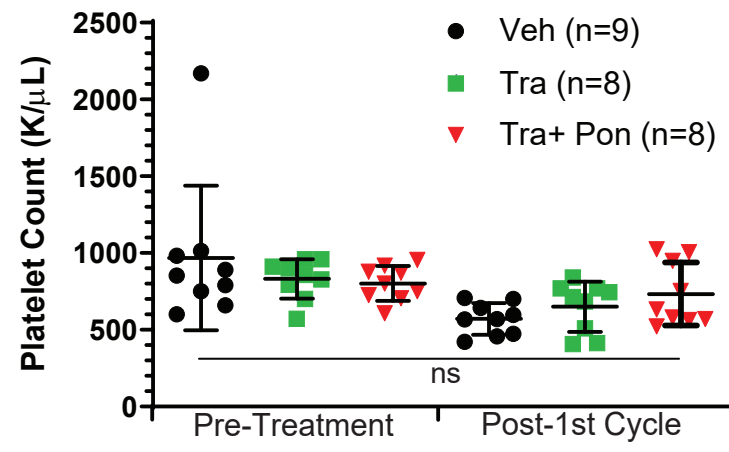

B

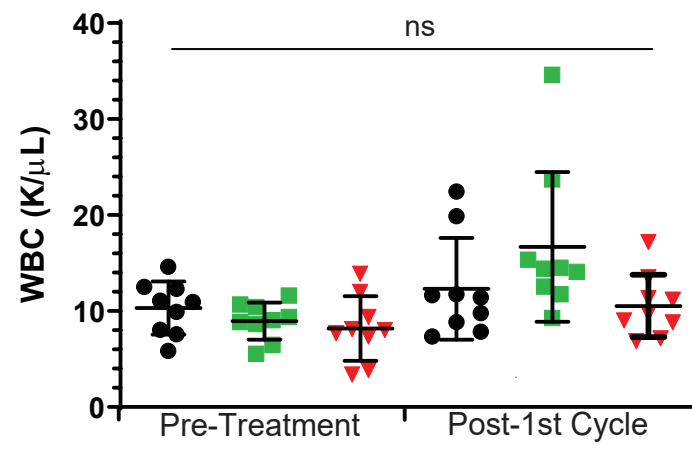

C

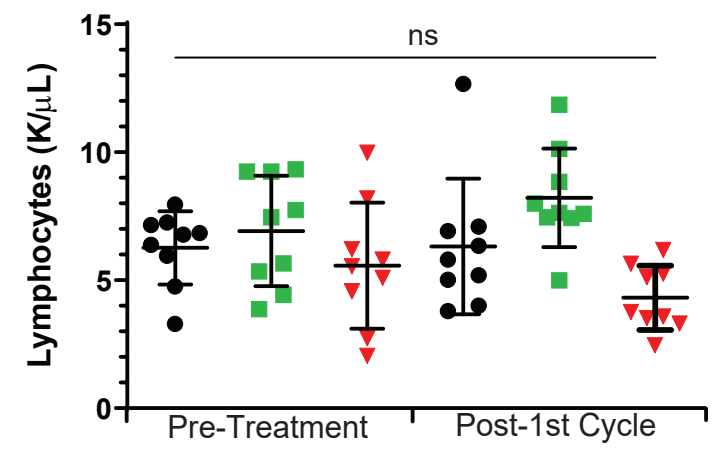

Supplement: Supplementary file 10 — Supplementary Information 10. [file 41598_2022_14114_MOESM10_ESM.pdf]
