# Supplementary material for: Ponatinib sensitizes myeloma cells to MEK inhibition in the high-risk VQ model
Source: Sci Rep. 2022 Jun 23;12:10616. doi: 10.1038/s41598-022-14114-z (PMC9226136; doi:10.1038/s41598-022-14114-z)
Supplement: Supplementary file 7 — Supplementary Information 7. [file 41598_2022_14114_MOESM7_ESM.pdf]

Figure S6-Zhang

A

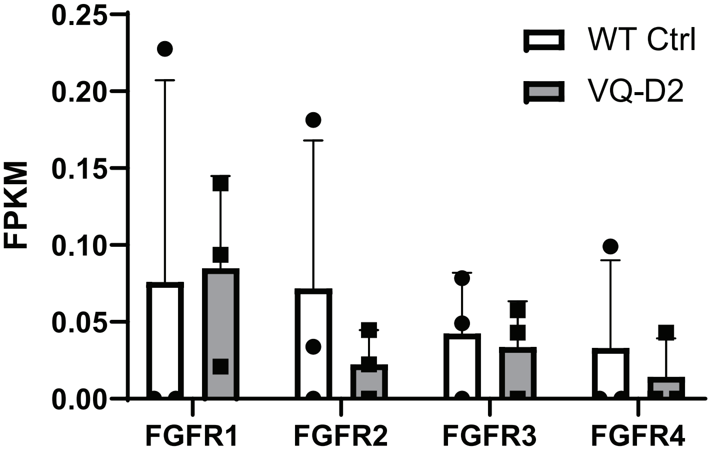

B

FGFRi	Relative Viability - 100nM	Relative Viability - 100nM + 10nM Tra	Relative Viability - 1000nM	Relative Viability - 1000nM + 10nM Tra
Sorafenib	1.23	0.52	1.12	0.49
Dovitinib	1.20	0.46	1.04	0.29
Pazopanib HCl	1.04	0.45	0.99	0.46
Lenvatinib	0.94	0.45	0.95	0.40
