# Supplementary material for: Ponatinib sensitizes myeloma cells to MEK inhibition in the high-risk VQ model
Source: Sci Rep. 2022 Jun 23;12:10616. doi: 10.1038/s41598-022-14114-z (PMC9226136; doi:10.1038/s41598-022-14114-z)
Supplement: Supplementary file 11 — Supplementary Information 11. [file 41598_2022_14114_MOESM11_ESM.docx]

#### Supplemental Information

**Supplemental Figure Legends**

**Figure S1. MEK inhibition via trametinib prevents CD8 T cell exhaustion in the VQ model.** CD45.1^+^ recipient mice were injected with 0.5x10^6^ VQ-D1 bone marrow cells. Once MM was established, mice were treated with 0.5mg/kg of Trametinib daily for 5 weeks. Mice were sacrificed and bone marrow and splenocytes were collected and analyzed by flow cytometry. (A) tSNE analysis of CD3+ cells from the bone marrow (BM) of treated VQ mice. Cells were gated on CD8 and expression of immune checkpoint receptors are highlighted. (B) Bar graphs shows frequency of exhausted (PD-1+TIGIT+LAG3+CD44+CD62L-) CD8 T cells among total CD8 T cells from BM and spleen of treated mice. Unpaired, two-sided was performed. *, p <0.05; **, p<0.01

**Figure S2. VQ MM cells do not respond to lenalidomide.** VQ 4935 and 4938 cells were treated with a wide range of concentrations of lenalidomide for 48 hours. Cell viability was then measured using the CellTiter-Glo assay. Relative viabilities to DMSO treated control are presented. IC_50_ values were calculated by logistic regression using the GraphPad Prism software.

**Figure S3. Validation of positive hits from AOD IX library using VQ 4935 and 4938 cells.** VQ 4935 and 4938 cells were treated with the indicated concentrations of panobinostat (A), romidepsin (B), vinblastine sulfate (C), and vincristine sulfate (D) for 48 hours. Cell viability was then measured using the CellTiter-Glo assay. Relative viabilities to DMSO treated control are presented. IC_50_ values were calculated by logistic regression using GraphPad Prism software.

**Figure S4. Proteasome inhibitors show short-term efficacy in the VQ model.** (A) Bortezomib treatment of VQ 4935 and VQ 4938 cells. Assay conditions are as described in Figures S2 and S3. (B-C) CD45.1^+^ recipient mice were injected with 0.5x10^6^ VQ-D1 bone marrow cells. Once MM was established, mice were treated with 0.5mg/kg of bortezomib via intraperitoneal (IP) injection twice a week. (B) Serum protein electrophoresis (SPEP) was performed to quantify the γ-globulin/Albumin (G/A) ratios in VQ recipient mice before treatment and at day 21 of treatment. (C) Kaplan-Meier survival curves were plotted against days after treatment. Log-rank test was performed. (D) Carfilzomib treatment of VQ 4935 and VQ 4938 cells. Assay conditions are as described in Figures S2 and S3. (E-F) CD45.1^+^ recipient mice were sub-lethally irradiated and injected with 0.5x10^6^ VQ-D1 bone marrow cells. Once MM was established, mice were treated with vehicle (Veh) or combined carfilzomib (Cfz) and dexamethasone (Dex) for two weeks, followed by daily treatment with trametinib (Tra) and GSK525762 (GSK) for one week. The three-week treatment cycles were separated by one week of rest. (E) Serum protein electrophoresis (SPEP) was performed to quantify the G/A ratios in VQ recipient mice before treatment and at day 21 of treatment. (F) Kaplan-Meier survival curves were plotted against days after treatment. Log-rank test was performed. **, p<0.01; ***, p<0.001. ns, not significant.

**Figure S5. Combination Index analysis shows synergy between trametinib and ponatinib** VQ 4935 (A) and 4938 (B) cells were treated with the indicated concentrations of trametinib (Tra) and ponatinib (Pon) for 48 hours. Cell viability was then measured using the CellTiter-Glo assay. Relative viabilities to DMSO treated control are presented. Combination Index (CI) analysis was carried out using Compusyn v1.0 software. CI<1 indicates synergism.

**Figure S6. Fibroblast growth factor receptor (FGFR) signaling does not play a role in VQ cell growth.** (A) Transcript levels of FGFR1-4 in CD138^+^ B220^-^ cells from control and VQ-D2 recipients. FPKM, Fragments Per Kilobase of transcript per Million mapped reads. (B) Viability results for VQ 4938 cells treated with 100nM or 1000nM of the indicated FGFR inhibitor as a single agent or in combination with 10nM trametinib for 48 hours. Relative viability was calculated by proportion of luminescence in indicated well to average luminescence of DMSO-treated control wells as measured using CellTiter-Glo.

**Figure S7. Evaluation of human myeloma cell lines with trametinib and ponatinib.** (A) Dose-response results for ponatinib against the indicated human myeloma cell lines (HMCLs). IC_50_ values were calculated by logistic regression using the GraphPad Prism software. (B) Left: Selected viability results for combination treatment of trametinib (Tra) and ponatinib (Pon) against the indicated HMCL. Right: ZIP synergy plots of Tra and Pon for the indicated HMCL. Zip Synergy scores were generated using the SynergyFinder online tool.

**Figure S8. Complete blood count results for VQ-D1 mice treated with 0.2mg/kg trametinib and 10mg/kg ponatinib.**

Complete blood count of peripheral blood samples collected from VQ-D1 recipient mice before treatment and at day 21 of treatment. Quantification of platelet (A), lymphocyte (B), and white blood cell (WBC) (C). Results are presented as mean + SD. **, p<0.01.

**Figure S9. Complete blood count results for VQ-D1 mice treated with 0.5mg/kg trametinib and 10mg/kg ponatinib.**

Complete blood count of peripheral blood samples collected from VQ-D1 recipient mice before treatment and at day 21 of treatment. Quantification of platelet (A), lymphocyte (B), and white blood cell (WBC) (C). Results are presented as mean + SD. ns, not significant.
