# Supplementary material for: Ponatinib sensitizes myeloma cells to MEK inhibition in the high-risk VQ model
Source: Sci Rep. 2022 Jun 23;12:10616. doi: 10.1038/s41598-022-14114-z (PMC9226136; doi:10.1038/s41598-022-14114-z)
Supplement: Supplementary file 12 — Supplementary Information 12. [file 41598_2022_14114_MOESM12_ESM.docx]

**Table S1 Relative Viability of representative drugs from NCI147 library**

| Drug Class | Drug | Relative Viability -100nM | Relative Viability -1000nM |
| --- | --- | --- | --- |
| Positive | Bortezomib | 0.01 | 0.01 |
|  | Carfilzomib | 0.02 | 0.02 |
|  | Ixazomib | 0.02 | 0.01 |
|  | Panobinostat | 0.03 | 0.02 |
|  | Romidepsin | 0.02 | 0.02 |
|  | Vincristine | 0.34 | 0.12 |
|  | Vinblastine | 0.31 | 0.06 |
|  | Dactinomycin | 0.02 | 0.03 |
|  | Plicamycin | 1.19 | 0.13 |
|  | Trametinib | 0.19 | 0.12 |
| False Negative | Thalidomide | 1.1 | 1.12 |
|  | Lenalidomide | 1.1 | 1.08 |
|  | Pomalidomide | 1.1 | 1.08 |
|  | Cyclophosphamide | 1.18 | 1.12 |
| Negative | Venetoclax | 1.12 | 1.03 |

Relative viability was calculated by proportion of luminescence in indicated well to average luminescence of DMSO-treated control wells as measured using CellTiter-Glo.
